# Transobturator Midurethral Slings versus Single-Incision Slings for Stress Incontinence in Overweight Patients

**DOI:** 10.1590/S1677-5538.IBJU.2014.0209

**Published:** 2015

**Authors:** Omer Bayrak, Ilker Seckiner, Gokhan Urgun, Haluk Sen, Caglayan Ozcan, Sakip Erturhan

**Affiliations:** 1 Department of Urology, University of Gaziantep, Gaziantep, Turkey; 2 Department of Obstetrics and Gynecology, University of Gaziantep, Gaziantep, Turkey

**Keywords:** Urinary Incontinence, Stress, Overweight, Suburethral Slings

## Abstract

**Purpose:**

To compare transobturator midurethral sling (TOS) and single-incision sling procedures in terms of their effects on urinary incontinence and the quality of life in overweight (BMI ≥25-29.9 kg/m2) female patients using the International Consultation on Incontinence Questionnaire scoring form (ICIQ-SF) and Quality of Life of Persons with Urinary Incontinence scoring form (I-QOL).

**Materials and Methods:**

In this prospective trial, the patients were divided into two groups consecutively; first 20 overweight female patients underwent the TOS (Unitape T®,Promedon, Cordoba, Argentina) procedure and the subsequent 20 consecutive overweight female patients underwent the single-incision sling [TVT-secur (Ethicon Inc., Sommerville, USA)] procedure. Age, urinary incontinence period, parity and daily pads usage were recorded. No usage of pads was defined as subjective cure rate postoperatively. Before the operation and 6. month after the surgery, the patients completed the ICIQ-SF and I-QOL.

**Results:**

There was no significant difference between the two groups in terms of mean age, duration of incontinence, parity, and BMI (p>0.05). ICIQ-SF and I-QOL revealed that the patients in the TOS group showed significantly better improvement (76.20% versus 64.10%, p=0.001, 81.31% versus 69.28%, p=0.001, respectively). In addition, subjective cure rates were found higher in TOS group (75% versus 55%, p=0.190).

**Conclusions:**

The existing data is showed that incontinence symptoms and the quality of life have higher improvement in overweight female patients who underwent the TOS procedure. It is likely that the TOS procedure may provide stronger urethral support and better contributes to continence in this group of patients.

## INTRODUCTION

The risk factors for urinary incontinence include age, menopause, parity, obesity, vaginal delivery, and hysterectomy (1–4). Especially, the effect of obesity on stress urinary incontinence (SUI) has been shown in many studies (5). The authors emphasized that intra-abdominal pressure increased in relation to increased weight, which in turn resulted in SUI (6). In the study by Thubert et al. in 2012, the relative risk of urinary incontinence was 5-fold higher in morbid obese women compared to women with a normal weight, and they reported a 50% decrease in the frequency of incontinence with a 10% decrease in the body weight (7).

Currently, sling surgical procedures (suburethral sling, vaginal wall slings, transvaginal tape, transobturator midurethral sling (TOS), single-incision sling) used in the surgical treatment of SUI are the first choice in most patients (8). The single-incision sling is a less invasive procedure and it has been developed to be an alternative to the TOS procedure. On the other hand, Moore RD et al. reported that the short size and smaller surface area of the single-incision sling compared to TOS had led to efficiency and safety concerns in overweight women (9).

The aim of the present study was to compare TOS and single-incision sling procedures in terms of their effects on urinary incontinence and the quality of life in overweight female patients using the International Consultation on Incontinence Questionnaire scoring form (ICIQ-SF) and Quality of Life of Persons with Urinary Incontinence scoring form (I-QOL).

## MATERIALS AND METHODS

After obtaining approval from the University Ethics Committee (protocol number: 03.04.2012/145), a total of 40 overweight women with stress urinary incontinence were included in the study. The participants signed an informed consent form that explained the primary and secondary outcomes of the study.

**Figure 1:**
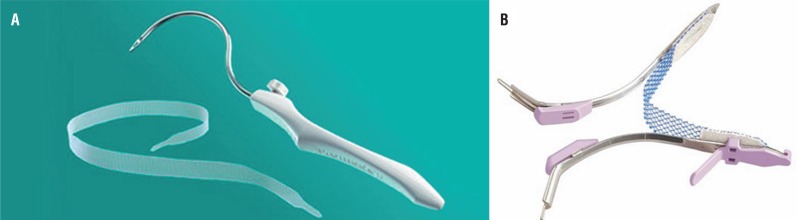
A) Transobturator midurethral sling: Unitape T®, Promedon, Cordoba, Argentina. B) Single-incision sling: TVT-secur, Ethicon Inc., Sommerville, USA.

**Figure 2:**
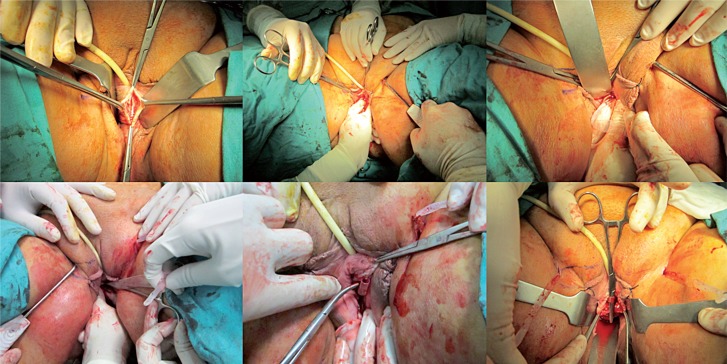
Transobturator midurethral sling surgery.

**Figure 3:**
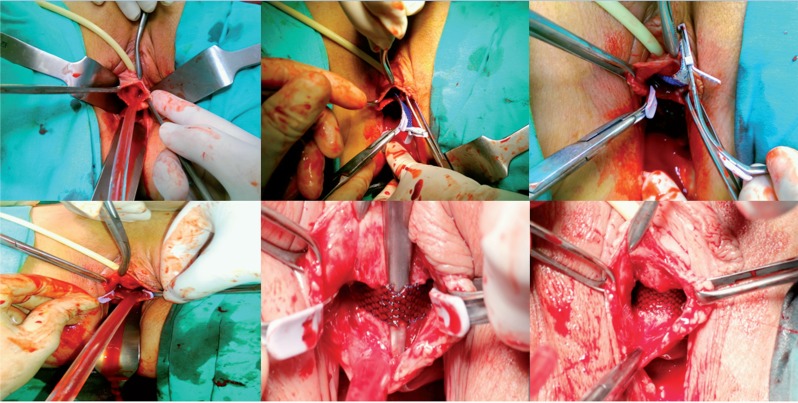
Single-incision sling surgery.

In this prospective trial, the patients were divided into two groups consecutively; first 20 overweight female patients underwent the TOS (Unitape T®, Promedon, Cordoba, Argentina) procedure and the subsequent 20 consecutive overweight female patients underwent the single-incision sling [TVT-secur (Ethicon Inc., Sommerville, USA)] procedure (Figures 1–3). Body mass index (BMI) 25-29.9 kg/m^2^ was accepted as overweight (10). Age, duration of urinary incontinence, parity and daily pads usage were recorded. All patients underwent physical and gynecological examinations, complete urine analysis, urine culture, Q-tip test, and urodynamic studies in the preoperative period. Daily pads usage was evaluated again at 6 months office visit. No usage of pads was defined as subjective cure rate (11).

Urodynamic evaluation was performed by the Medical Measurements System (MMS UD-2000, Enschede, the Netherlands). Throughout the cystometry filling period, especially at 200mL, it was asked the patient to cough. If there was any involuntary leakage, Valsalva Leak Point Pressure (VLPP) was recorded. These provocative activities were repeated at every 100mL. SUI was defined as having urinary incontinence due to the increase in abdominal pressure without detrusor contraction. Patients with VLPP below 60cmH_2_0 were evaluated as internal sphincter deficiency (ISD), VLPP above 90cm H_2_0 as urethral hypermobility, and VLPP between 60-90cmH_2_0 as mix pathology (12).

The present study included patients with stress urinary incontinence aged above 18 years who had complaints for more than 1 year, and in whom urodynamic tests showed SUI and the cough stress test in the standing position was positive (9). Patients with stress urinary incontinence accompanied with neurogenic bladder component, urinary tract infections, pregnant women, patients with a history of incontinence surgery, history of pelvic radiotherapy, pelvic organ prolapse stage ≥3, urge incontinence, residual urine volume above 100 cc, and patients with urethral hypomobility (Q-tip ≤20) were excluded from the study. TOS and single-incision sling procedures were performed by two surgeons who operated approximately 20 patients per year for sling surgeries.

### 

#### Scoring Forms

Before the operation and sixth month after the surgery, the patients completed the ICIQ-SF and I-QOL scoring forms for the assessment of urinary incontinence and the quality of life, respectively. Turkish versions of the above-mentioned forms have been previously validated for use in Turkey.

The validity study for the Turkish version of ICIQ-SF was initiated by the translation of the scoring form by a native Turkish speaker with a high proficiency in English in line with the recommendations of Kerry Avery, a member of the ICIQ Development group. The scores were calculated according to the answers given to these questions (13): “How often do you leak urine? (0-5 points),” “Usually, how much do you leak urine (whether you wear protection or not)? (0-6 points),” “Overall, how much does leaking urine interfere with your daily life? (0-10 points).”

The Turkish translation of the 22-item I–QOL questionnaire was developed by Bushnell DM, Martin M, and Patrick D for assessing the quality of life. It was applied to patients after the Turkish version was validated for usage in Turkey (14, 15). This questionnaire represented a reliable scale with high sensitivity in the assessment of the quality of life. Using this questionnaire, the patients were asked to subjectively rate how much they felt uncomfortable about their condition over a 10-point scale (0 points indicate no discomfort and points below 10 indicate discomfort).

### Statistical analysis

The statistical software package, SPSS 11 for Windows, was used in statistical analyses, and the data were expressed as arithmetic average and standard deviation. Chi-square distribution test was used to analyze categorical data, and Mann-Whitney U-test was used to compare average values. P value <0.05 was considered statistically significant with 95% confidence interval.

## RESULTS

Mean age was 53.25±5.70 (44–63) years in the TOS group and 52.20±5.81 (43-62) years in the single-incision sling group. There was no significant difference between the two groups in terms of mean age, duration of incontinence, parity, and mean body mass index (p>0.05) (Table- 1). During the urodynamic evaluation, ISD was determined in 1 one patient in each group, and urethral hypermobility was observed in all other patients.

**Table 1:** Demographic data of the patients and the operations.

	Transobturator Sling	Single-Incision Sling	p value
Age (year)	53.25±5.70	52.20±5.81	p=0.565
BMI (kg/m^2^)	27.75±1.29	28.40±0.99	p=0.201
Parity (n)	3.95±1.66	3.85±1.59	p=0.779
Incontinence period (year)	3.45±0.94	4.10±1.07	p=0.720
Operation time (minute)	24.25±3.41 (18–28)	16.30±1.65 (14–20)	p=0.0001
Intraoperative complications	-	Bladder perforation in 1 (5%) patient	p=0.311
Postoperative complications	‘de novo’ urge incontinence in 1 (5%) patient	‘de novo’ urge incontinence in 1 (5%) patient	p=1

Preoperatively, mean daily pad usage was calculated as 4.45±0.82 (3–6) in the TOS group, and 4.25±0.78 (3–6) in the single-incision sling group (p=0.495). Postoperatively, no pad usage was observed in 15 female patients in TOS group and in 11 female patients in the single-incision sling group at 6 months follow-up, therefore subjective cure rates were calculated as 75% and 55%, respectively (p=0.190) (Table-2).

**Table 2:** Data of pad usage.

	Transobturator Sling	Single-Incision Sling	p value
Preoperative daily pads usage (mean)	4.45±0.82 (3-6)	4.25±0.78 (3-6)	p=0.495
Postoperative daily pads usage (mean)	0.30±0.57 (0-2)	0.55±0,68 (0-2)	p=0.192
Postoperatively, the number of patients with	15 / 20	11 / 20	p=0.190
no pad usage (n / all patients)			
Subjective cure rates	75%	55%	p=0.190

The comparison of scores achieved in ICIQ-SF administered before and 6 months after the surgery revealed that the patients in the TOS group showed significantly better improvement when compared to patients in the single-incision sling group (76.20% versus 64.10%, p=0.001). Similarly, the comparison of scores achieved in I–QOL administered before and 6 months after the surgery revealed that the patients in the TOS group showed significantly better improvement (81.31% versus 69.28%, p=0.001) (Table-3).

**Table 3:** Pre-operative and post-operative ICIQ-SF and I-QOL scores.

	Transobturator Sling	Single-Incision Sling	p value
Preoperative ICIQ-SF scores (mean)	16.60±2.11	17.55±2.25	p=0.165
Postoperative 6.month ICIQ-SF scores (mean)	3.95±1.43	6.30±1.59	p=0.001
Improvement on ICIQ-SF scores	76.20%	64.10%	p=0.001
Preoperative I-QOL scores (mean)	61.00±8.44	63.65±12.02	p=0.369
Postoperative 6. month I-QOL scores (mean)	11.40±4.36	19.55±7.02	p=0.001
Improvement on I-QOL scores	81.31%	69.28%	p=0.001

Mean operation time was 24.25±3.41 (18–28) minutes in the TOS group and 16.30±1.65 (14–20) minutes in the single-incision sling group (p<0.05). In both groups, Foley urinary catheters were withdrawn at 24 hours after surgery. None of the patients had urinary retention or required re-catheterization. Bladder perforation occurred in one patient in the single-incision sling group intra-operatively. Urine flow was observed while advancing sling material attached to the forceps in the right para-urethral area. The mesh was removed and cystoscopy was performed. Five milimeter perforation site was observed at the junction of the base and right lateral wall of the bladder. The perforation site was primarily repaired via the transvaginal approach. Single-incision sling material was then placed after performing para-urethral-paravaginal dissection. In the postoperative period, the urinary catheter was left in the place for 7 days, and the patients were then discharged with complete recovery.

At postoperative 6 months, none of the patients developed urinary infection, mesh erosion, hematoma, abscess, urinary retention, or leg pain. “Denovo” urgency and urge incontinence occurred in 1 patient in the TOS group and 1 patient in the single-incision sling group during postoperative follow-up period. Two patients received solifenacin succinate therapy and both responded well to the treatment.

## DISCUSSION

Sling operation is one of the oldest methods performed successfully until today in the treatment of urinary incontinence. The procedure was conducted for the first time by Goebell (16) in 1910, and sling material was placed on the bladder neck. The first series of single-incision sling was presented by Smith et al., at the International Urogynecology Congress held in 2008. This study was launched in 2002 and authors reported good tolerance to local anesthesia, low morbidity, and early return to daily activities, after a mean of 2 year follow-up (17).

After this presentation, various studies have been published reporting subjective and objective outcomes of the several single-incision sling materials, and the studies reported a success rate ranging from 63% to 97% in short follow-up period of 3 months (18–20). In a prospective observational study of 25 patients, Cornu et al. reported a 94% success rate in the short term, although the success rate had later declined to 40% in long term follow-up period of 30.2 months (21). Likewise, North et al. reported 10% success rate at the end of a two year follow-up (22). The major reason underlying the low success rate in the long term was suggested to be the low resistance of the sling tips that provide attachment to the obturator membrane or muscles, inability to appropriately adjust the tension of the material after placement, and the difficulty of insertion-fixation mechanisms (23). In order to reach the success rate obtained by other sling methods, Oliviera et al. suggested that the mesh should be tilted back on the urethra until the underlying tissue becomes visible through the mesh and the tension should be well adjusted (24).

There is an emphasis on obesity as one of the factors negatively affecting success rate of the incontinence surgery (25). Intra-abdominal pressure increases with the increase in the body weight, and this may affect the strength of pelvic floor by inducing nerve and muscle injuries (26). In a study by Meschia et al., 206 patients were divided into three groups as normal weight (BMI <25kg/m^2^), overweight (BMI 25-29.9kg/m2), and obese (BMI≥30kg/m^2^); the success rate was found to be higher in the normal weight group compared to obese patients (91.3% versus 75%, p=0.049) (8). They emphasized that the evaluation of the ICIQ-SF and Patient Global Impression of Improvement scores were stated to have significantly lower improvement in obese patients when compared to normal or overweight patients. On the other hand, Moore et al. performed MiniArc single-incision sling procedure in 68 obese (body mass index >30kg/m^2^) and 126 non-obese patients (<30kg/m^2^). They did not observe significant differences between the groups in terms of their score in cough stress test (81% obese vs. 86% non-obese; p=0.449), and median scores in Urogenital Distress Inventory 6 and Incontinence Impact Questionnaire 7 (p=0.126 and p=0.087, respectively) (9).

In a study considering prognostic factors and success rates while using the TOS procedure, Esin et al. divided the patients into two groups as obese (BMI≥30kg/m^2^) and non-obese (BMI<25kg/ m2) patients, and they compared the quality of life scores, urodynamic results, and objective cure rates between the groups. They reported that complete recovery and/or improvement in the symptoms was achieved both in obese and non-obese patients, and the quality of life improved in the postoperative period. They also emphasized that BMI did not affect the clinical effectiveness of TOS in the treatment of SUI (27). In the present study, TOS and single-incision sling procedures were compared in overweight female patients. The improvement in incontinence and the quality of life according to scores in ICIQ-SF and I-QOL were determined as 76.20% and 81.31% in the TOS group and 64.10% and 69.28% in the single-incision sling group. At postoperative 6 months follow-up, the scores were observed significantly higher in the TOS group (p<0.05). Also subjective cure rates were found higher in TOS group calculated by daily pad usage (75% versus 55%, p=0.190), although it was not statistically significant. These results suggest that TOS may be the first choice in the treatment of overweight patients.

Single-incision sling procedures involving a single incision have been designed to provide a less invasiveness and to reduce the damaging neural and vascular structures in the neighborhood retropubic space and obturator foramen while placing sling materials (28). However, variable complication rates have been reported while positioning sling material. Gauruder-Burmester et al. published the outcomes in 95 patients who underwent the single-incision sling procedure, and they reported bladder perforation in one patient (29). In the present study, bladder perforation occurred in one patient in the single-incision sling group, and the 5mm perforation site was repaired via the transvaginal approach. In addition, sling operations bring the risks of prolonged urinary retention, de novo urge incontinence, and mesh erosion. Postoperative de novo urgency was reported in 11% of the patients after retropubic suspension operation and in 7% of the patients after sling operation (30, 31). In the present study, “de novo” urgency and urge incontinence occurred in one patient in each group (5%), and were treated with solifenacin succinate successfully.

The limitations of our study are the small sample size, not inclusion of other BMI groups, and the short follow-up duration. We evaluated subjective cure rates by the number of daily pad usage. Probably pad-test might provide us more objective information. The current results need to be confirmed in prospective, randomized studies with larger patient series and long-term follow-up.

## CONCLUSIONS

The existing data showed that incontinence symptoms and the quality of life have higher improvement in overweight female patients who underwent the TOS procedure. Also subjective cure rates observed are higher in TOS group. It is likely that the TOS procedure may provide stronger urethral support and better contributes to continence in this group of patients.
